# Primary “Brown Pigment” Bile Duct Stones

**DOI:** 10.1155/1991/76160

**Published:** 1991

**Authors:** Luis Vitetta, Avni Sali, Peter Little, Jack Nayman, Ayman Elzarka

**Affiliations:** Department of Surgery University of Melbourne Repatriation General Hospital Heidelberg Victoria 3081 Australia

## Abstract

Bile duct stones from 42 patients were morphologically and chemically analysed. The calculi from 27 patients had important primary bile duct stone (PBDS) features, consisting of a general ovoid shape and fragile structure, with alternating light and dark brown pigmented layers on cross-section. Chemically these stones contained low levels of cholesterol, with high levels of bilirubin and calcium. Subsequent infrared spectroscopy analysis showed that calcium bilirubinate and calcium palmitate were the only calcium salts present. Calcium palmitate was prominent in the light brown layers. A morphological and chemical comparison with gallbladder stones showed that bile duct “stasis stones” were similar in morphological and chemical composition to the brown pigment gallbladder calculi. However, they were distinct from most gallbladder stones, indicating that primary bile duct calculi have an aetiology that is different to 90% of gallbladder calculi. Primary bile duct calculi were observed to occur with or without the presence of a gallbladder, and more interestingly, in the bile duct of two patients with cholesterol gallbladder stones. Bile duct bile of patients with primary choledocholithiasis were always moderately to profusely infected and with abundant calcium bilirubinate precipitation. Moreover, this study has shown that PBDS chemical analyses profiles were consistent and correlated well with their defined morphology. Consequently, PBDS may be accurately identified at the time of operation by morphology. An important aetiological factor would appear to be infection, which would seem to promote bile duct bile stasis and eventual stone growth.


*HPB Surgery*, 1991, Vol. 4, pp. 209–222
Reprints available directly from the publisher
Photocopying permitted by license only

© 1991 Harwood Academic Publishers GmbH
Printed in the United Kingdom

### PRIMARY "BROWN PIGMENT" BILE DUCT STONES

LUIS VITETTA,* AVNI SALI, PETER LITTLE, JACK NAYMAN and AYMAN ELZARKA

*Department of Surgery, University of Melbourne, Repatriation General Hospital, Heidelberg, Victoria, Australia*

*(Received 6 February 1991)*

Bile duct stones from 42 patients were morphologically and chemically analysed. The calculi from 27 patients had important primary bile duct stone (PBDS) features, consisting of a general ovoid shape and fragile structure, with alternating light and dark brown pigmented layers on cross-section. Chemically these stones contained low levels of cholesterol, with high levels of bilirubin and calcium. Subsequent infrared spectroscopy analysis showed that calcium bilirubinate and calcium palmitate were the only calcium salts present. Calcium palmitate was prominent in the light brown layers. A morphological and chemical comparison with gallbladder stones showed that bile duct "stasis stones" were similar in morphological and chemical composition to the brown pigment gallbladder calculi. However, they were distinct from most gallbladder stones, indicating that primary bile duct calculi have an aetiology that is different to 90% of gallbladder calculi. Primary bile duct calculi were observed to occur with or without the presence of a gallbladder, and more interestingly, in the bile duct of two patients with cholesterol gallbladder stones. Bile duct bile of patients with primary choledocholithiasis were always moderately to profusely infected and with abundant calcium bilirubinate precipitation. Moreover, this study has shown that PBDS chemical analyses profiles were consistent and correlated well with their defined morphology. Consequently, PBDS may be accurately identified at the time of operation by morphology. An important aetiological factor would appear to be infection, which would seem to promote bile duct bile stasis and eventual stone growth.

KEY WORDS: Calcium bilirubinate, calcium palmitate, composition, morphology, primary bile duct stones

### INTRODUCTION

In 1924 Aschoff[1] described in detail, the morphological features of a "primary biliary stasis stone", whose origin was in the bile ducts. Since then, the concept of the stasis stone or brown pigment stone in the bile ducts has developed into a distinct entity, with specific anatomical locus and pathology. Its pathogenesis remains controversial.

Animal models[2,3] have demonstrated that common duct and hepatic/intrahepatic duct stones form as a result of common duct obstruction and stasis. It has been suggested that common bile duct calculi result from chemical changes in the bile composition[4,5] with or without infection[2,3]. Mechanical aberrations, such as a diverticulum of the common duct, long cystic duct remnants and suture material from previous surgery, all favour the formation of primary bile duct stones[4,6].

*Address for correspondence: Dr. L. Vitetta, University Department of Surgery, Repatriation General Hospital, Heidelberg, Victoria 3081, Australia.

Abnormal functional dilation of the bile duct, giving rise to stasis, could also result in common bile duct stone production[4,5].

Primary bile duct stones (PBDS) are those which form in the bile duct as distinct from secondary bile duct stones (SBDS) which migrate from the gallbladder. PBDS have features that were initially described by Aschoff[1] in 1924. More recently, these stones have been referred to as the true primary bile duct stone, a stone originating in the same area in which it is found, that is, in the bile duct[4].

Primary bile duct stones usually occur singly, are dark brown and although of variable size, usually conform to the contours of the bile duct[7,8]. They can be crushed easily between the fingers to form "biliary mud"[9]. Although there have been many descriptions of bile duct stones, there is little information on the chemical composition and structure of PBDS. It has been reported that there are differences between PBDS and gallbladder stones in terms of cholesterol and calcium[10,11]. PBDS were found to have less than 25% by weight cholesterol supporting the contention that different aetiologic factors were involved in their formation. Our group has also shown that PBDS also contain high levels of bilirubin and calcium[10,12]. Controversy still exists as to what actually constitutes a primary or secondary bile duct calculus. This report has investigated the physical and chemical features of these calculi compared to those of the gallbladder. Cholesterol, bilirubin and calcium levels comprised the chemical profile in conjunction with the infrared spectroscopy data. These results were related to the morphological characteristics of the various stones types. Moreover, important aetiological factors such as bacterial infection of bile duct bile, calcium bilirubinate precipitation are suggested as being directly related with the pathogenesis of PBDS.

### MATERIALS AND METHODS

### *Calculi*

Bile duct calculi were obtained from 42 patients from a series of 937 patients undergoing cholecystectomy. Twenty-eight patients had not had a previous cholecystectomy, whereas the other 14 patients had a cholecystectomy and operative cholangiogram less than 1-12 years previously (Table 1).

### *Morphology*

Bile duct stones were examined for gross morphology by noting surface features, texture and cross-sectional appearance. "Soft" stones were defined as those which could easily be crushed into a coarse powder, "hard" were those which were not readily crushable between the fingers.

### *Chemistry*

Bile duct stone samples were chemically analysed for cholesterol bilirubin and calcium. All reagents for the chemical analysis were of analytical grade. Prior to analysis all stone samples were washed with distilled water, air dried, pulverized and then chemically analysed. Briefly, a fraction of the gallstone or bile duct stone

**Table 1** Bile Duct Stones

| Patient Type | *Stone Type** *PBDS* *No.* | *SBDS* *No.* |
|---|---|---|
| 1. No previous cholecystectomy | | |
| a) BDS only | 12 | 1 |
| b) BDS and GS | 4 | 10 |
| c) BDS and GB carcinoma | 1 | |
| 2. A previous cholecystectomy | | |
| a) Less than one year post cholecystectomy (via Dormia basket) | 0 | 2 |
| b) 2–12 years post cholecystectomy (via a second operation) | 10 | 2 |
| Totals | 27 | 15 |

* Subdivided according to morphological appearance.
BDS = bile duct stones, GS = gallstones, GB = gallbladder.

sample (usually 10 mg of powder) was extracted with acidified choloform:methanol (2:1) mixture. The resultant extract was subjected to cholesterol determination by the Boehringer-test combination cholesterol/catalase method as adapted by Roschlau[13]. Bilirubin was assayed by diazo-reaction with a modification of the method of Malloy and Evelyn as adapted by Henry *et al*[14]. Calcium was estimated by the method of Nakayama[15]. A 10 mg sample of gallstone or slude powder was ashed by heating (180°C) with concentrated sulphuric acid for two hours. Calcium was then determined by atomic absorption spectroscopy using a standard addition technique.

Gallstone samples from each major type and PBDS and SBDS were qualitatively analysed by infrared spectroscopy, which was used to confirm the presence of the common gallstone components, namely cholesterol, bilirubin and calcium, the latter in the form of calcium bilirubinate, calcium carbonate, calcium palmitate, and calcium phosphate. Reference infrared spectra of cholesterol, calcium carbonate (CaCarb), and calcium phosphate (CaPhos), were recorded on high purity samples (Sigma). Calcium bilirubinate (CaB), and calcium palmitate (CaPalm) were prepared according to a previously described method[19].

Aliquots (1 mg) of desiccated stone powder were finely ground with an agate mortar and pestle, and then mixed and reground with 100 mg of spectral grade potassium bromide. The resulting fine homogenous powder mix was placed in a stainless steel die and pressed at 172.5 MPa in a hydraulic press for five minutes. An infrared spectrum was obtained on the wafer with a Perkin-Elmer 457 grating infrared spectrophotometer. The method was that adapted from Soloway *et al* (1977)[16].

### *Bile Culture*

Wherever bile samples from the bile duct were available they were examined for bacteria by Gram's stain. Fifteen samples were obtained from patients undergoing a cholecystectomy and bile duct exploration, and five from patients with a previous cholecystectomy. Bile was directly plated onto sheep blood agar (SBA). McConkey's agar, horse blood agar (HBA), lysed horse blood with Vancomycin and Vitamin K (LKV), Nagler's agar, phenylethyl alcohol agar and cooked meat medium were incubated anaerobically in jars flushed with a commercial gas mixture (85% $N_2$, 5% $H_2$, 10% $CO_2$). All plates were incubated at 35°C for five days and examined daily for growth. Bacterial growth was measured as a function of colony forming units (CFU) per mililitre of bile. Scant growth was less than 1000 CFU/ml, moderate 10,000-100,000 CFU/ml and profuse growth greater than 100,000 CFU/ml.

### *Bile Microscopy*

Bile samples from the bile duct were also examined for biliary crystals (cholesterol [ChC]) and CaB within five minutes of aspiration. Biles were mixed thoroughly and placed in test tubes in a water bath at 37°C, examined microscopically in direct and polarized light at low ($\times 100$) and high ($\times 400$) magnifications.

### RESULTS

### *Morphology*

The bile duct stones from 27 patients were classified as PBDS from their morphology (Table 1). These stones were soft and easily fragmentable (Figures 1 and 2). They were dark brown on the surface and were either smooth or rough, mostly with an ovoid shape. The stones occurred singly or as multiples, in which case they were also observed to be facetted (Figure 3). The bile duct stones from 14 patients were however, classified as secondary bile duct stones. The stones from 12 patients were hard, white to pale yellow in colour and highly facetted. Cross-sectioning these calculi revealed light yellow radial striations with small brownish nuclei, characteristics which are common among cholesterol calculi of the gallbladder. The calculi from two patients were observed to be of the brown pigment type with mixed layers of white/yellow and pigment matter on cross-section. Furthermore, a single bile duct stone from one patient was found to be very distinct on cross-section. It had a hard pale core (32% total stone weight) and a softer brown shell (68% of total stone weight) (Table 2).

### *Chemistry*

The "soft" bile duct stones from 27 patients contained significantly high levels of bilirubin with a mean (SD) of 21.6% (8.5) and a mean calcium (SD) level of 5.5% (4.8). However, their mean cholesterol content was found to be very low with a mean (SD) of 22.9% (11.4). Furthermore, the stones from 20 of these patients had a cholesterol content of 25% or less (Table 3). The "hard" bile duct stones from 12 patients were cholesterol rich with a mean (SD) of 92.5% (6.6) and with low levels of bilirubin and calcium with means (SD) of 1.3% (0.5) and 1.5% (1.3) respectively (Table 2). The calculi from two other patients had means (SD) of cholesterol 62.9% (3.8), bilirubin 13.9% (2.2) and calcium 5.8% (3.8).

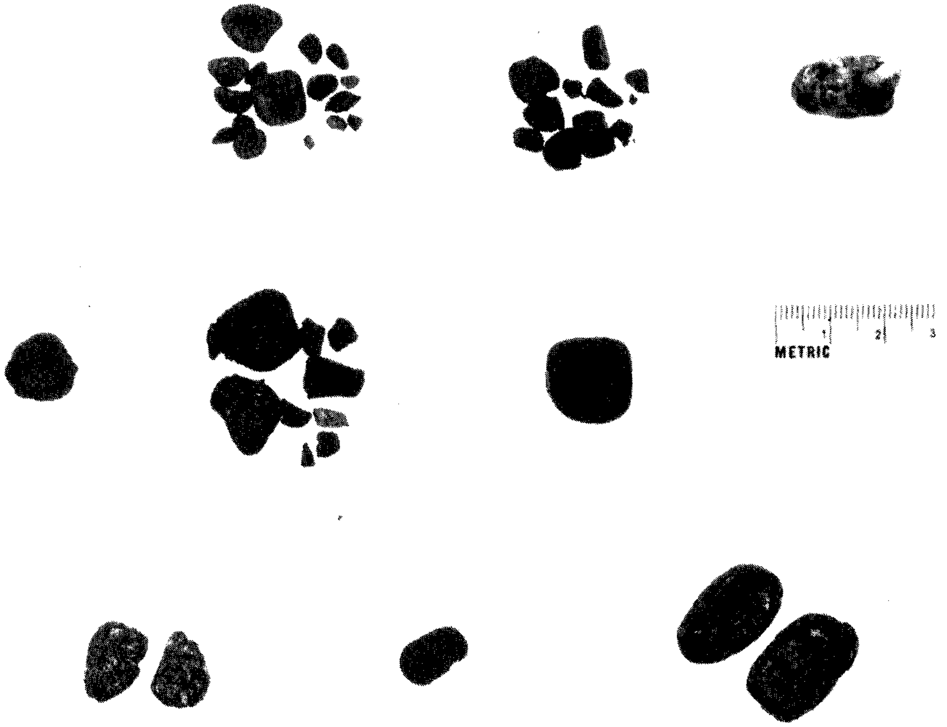

**Figure 1** PBDS samples.

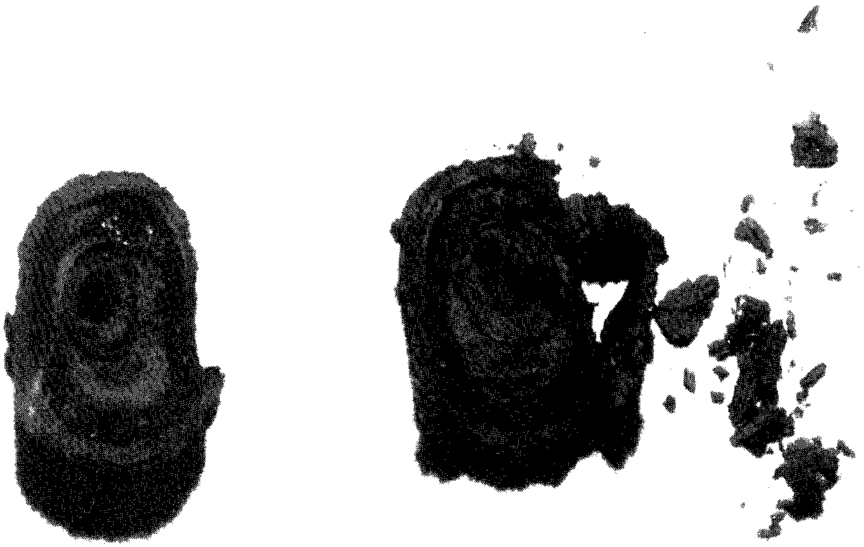

**Figure 2** PBDS cross-section view. Note layered appearance.

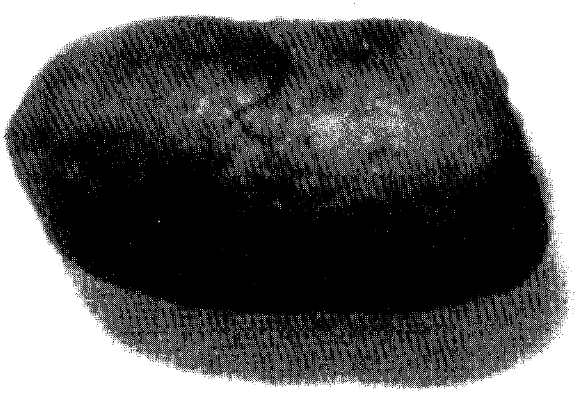

**Figure 3** Classical appearance of PBDS. Note ovoid shape.

**Table 2** Bile duct stone composition.

| Gallstone Types | PBDS | SBDS | | Combination Stone | | |
|---|---|---|---|---|---|---|
| Cases (number) | (27) | (14) | (1) | | | |
| Stone Subtypes | — | Cholesterol | Brown Pigment | Core | Shell | Core + Shell |
| Number | | 12 | 2 | | | |
| Composition Mean (SD) (Range) | | % Of Dry Weight of Sample | | | | |
| Cholesterol | 22.9(11.4) (<1–49.2) | 92.5(6.5) (79.0–98.0) | 62.9(3.8) (59.0–68.0) | 99 | 64 | 80 |
| Bilirubin | 21.6(8.5) (3.3–38.0) | 1.3(0.5) (<1–2.0) | 13.9(2.2) (11.0–16.0) | <1 | 18 | 11 |
| Calcium | 5.5(4.8) | 1.5(1.3) | 5.8(3.8) | <1 | 2 | 1.5 |
| | | IR Spectroscopy Detected Cases (No.) | | | | |
| Calcium bilirubinate | (27) | (7) | (2) | (nd) | + | + |
| Calcium carbonate | (nd) | (5) | (nd) | (nd) | (nd) | (nd) |
| Calcium palmitate | (27) | (nd) | (2) | (nd) | + | + |
| Calcium phosphate | (nd) | | | | | |
| Cholesterol | (26) | (12) | (2) | + | + | + |

nd = not detected
+ = detected

**Table 3** Individiual PBDS percentage dry weight compositions

| | Pre-cholecystectomy (17 Cases) Without GBL (12 cases) | | | With GBL stones (4 Cases) | | | GBL Carcinoma (1 Case) | | | Post-cholecystectomy (10 cases) | | |
|---|---|---|---|---|---|---|---|---|---|---|---|---|
| | *Ch* | *Br* | *Ca* | *Ch* | *Br* | *Ca* | *Ch* | *Br* | *Ca* | *Ch* | *Br* | *Ca* |
| | 20.0 | 23.3 | 3.1 | 1.0 | 38.0 | 5.3 | 14.0 | 21.0 | 2.0 | 17.2 | 3.3 | 4.0 |
| | 14.0 | 4.0 | 12.0 | (89.5)* | (<1) | (1.0) | | | | 19.1 | 25.2 | 5.1 |
| | 13.1 | 15.3 | 2.9 | | | | | | | 11.3 | 6.2 | 2.1 |
| | 18.3 | 17.5 | 4.2 | | | | | | | 23.0 | 22.0 | 9.0 |
| | 14.1 | 21.0 | 2.0 | 15.1 | 32.2 | 2.2 | | | | 49.2 | 18.1 | 5.2 |
| | 25.4 | 31.3 | 5.0 | (96.5) | (<1) | (<1) | | | | 32.2 | 18.9 | 12.2 |
| | 18.6 | 26.5 | 2.1 | 23.1 | 27.4 | 2.2 | | | | 28.9 | 28.1 | 10.1 |
| | 16.3 | 29.2 | 2.3 | (78.5) | (1.5) | (<1) | | | | 21.5 | 21.5 | 11.5 |
| | 23.7 | 19.2 | 1.3 | | | | | | | 15.5 | 15.0 | 5.0 |
| | 38.3 | 34.2 | 23.6 | 45.9 | 16.3 | 4.1 | | | | | | |
| | 44.1 | 23.0 | 3.0 | (75.5) | (1.0) | (1.5) | | | | | | |
| | 22.0 | 21.1 | 3.1 | | | | | | | | | |
| Mean | 22.3 | 22.1 | 4.9 | 21.3 | 28.5 | 3.5 | | | | 25.2 | 18.4 | 6.9 |
| (SD) | (9.7) | (8.0) | (6.6) | (18.8) | (9.2) | (1.5) | | | | (11.2) | (8.2) | (3.5) |

*GBL* = gallbladder; *Ch* = cholesterol; *Br* = bilirubin; *Ca* = calcium; * = gallstone samples.

A single bile duct stone with a hard core/soft shell from one patient, had a core which was basically pure cholesterol (99%), with bilirubin and calcium levels of less than 1% respectively. Its shell consisted of 68% cholesterol, 18% bilirubin and 2% calcium. This was classified as a combination stone. Previously it has been estimated that approximately 80% of gallbladder calculi at cholecystectomy are of the cholesterol type and that the remaining 20% constitute the brown and black pigment variety[18]. A comparison of chemical compositions between PBDS, SBDS and all gallstone types[18] showed that PBDS had a similar chemical composition to that of the brown pigment gallbladder stones (Tables 2, 3 and 4). Moreover, bile duct stones thought to be primary from pre-cholecystectomy patients were similar in chemical composition to those PBDS from post-cholecystectomy patients (Table 3).

### *Infrared Spectroscopy*

Infrared spectroscopy analysis confirmed the presence of the main constituents of gallstones, namely cholesterol, bilirubin and calcium. Infrared spectroscopy showed that all PBDS contained calcium bilirubinate and calcium palmitate (Figure 4).

Infrared spectroscopy analysis of SBDS (from 14 patients) showed very clear and sharp cholesterol bands. Calcium bilirubinate was only rarely observed in these samples, whereas calcium carbonate was observed in 50% of the samples. Infrared spectra of the shell and core areas of the combination stone gave distinct spectral patterns (Figure 4). The core was observed to be almost pure cholesterol, whereas the shell, in addition to containing cholesterol, contained calcium in the form of calcium bilirubinate and calcium palmitate. Comparisons were made with an array

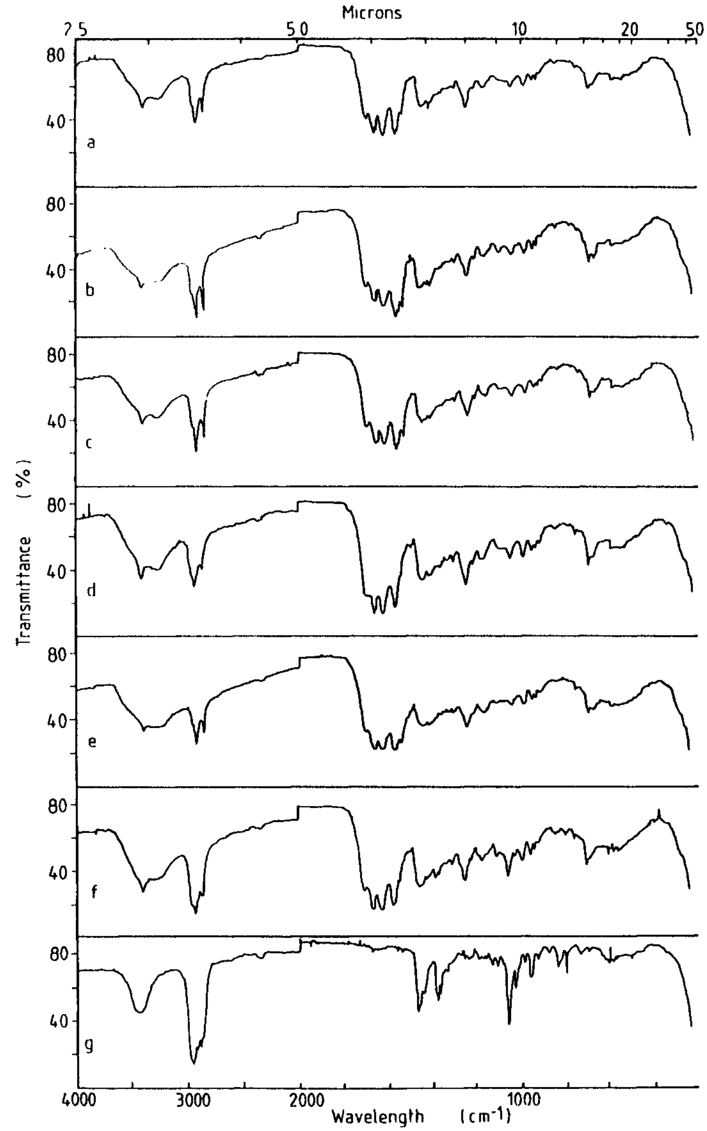

**Figure 4** IR spectra of PBDS. a–b: pre-cholecystectomy group. c–e: post-cholecystectomy group. f: shell. g: core, combination stone.

of gallstones, that consisted of single, multiple cholesterol, brown and black pigment samples.

Morphologically, PBDS were distinct from all gallstones. Infrared spectroscopy confirmed that PBDS and brown pigment gallbladder stones had a similar infrared pattern, showing that both contained calcium bilirubinate and calcium palmitate. The infrared patterns of PBDS were very distinct though to all other gallbladder stone types[18].

**Table 4** Gallstone Types and Composition (From a Series Previously Described in our Department)[18]

| *Gallstone Types (No.)* | | *% Dry Weight (Mean SD)* *Cholesterol* | *Bilirubin* | *Calcium* |
|---|---|---|---|---|
| CHOLESTEROL | Single (19) | 92.1 (6.1) | $<1$ | 2.1 (1.7) |
| | Multiple (216) | 87.5 (8.3) | $<1$ | 1.4 (1.4) |
| PIGMENT | Brown (42) | 61.2 (10.5) | 13.6 (6.6) | 5.8 (2.5) |
| | Black (28) | 18.1 (8.7) | 13.7 (6.7) | 10.3 (6.6) |

### *Bile Culture*

The extent of bacterial growth that was observed in bile duct bile ranged from scant ($<10^3$ CFU/ml) to moderate ($10^4$–$10^5$ CFU/ml) to profuse ($>10^5$ CFU/ml). Of 20 bile duct biles tested, 9 (45%) had a positive culture. Two, four and three bile samples respectively had scant, moderate and profuse bacterial growth. The most common bacterial species cultured was Escherichia coli. Other bacterial species cultured consisted of Klebsiella oxytoca, Klebsiella pneumoniae citrobacter freundii, clostridium perfringens, bacteroides fragilis.

### *Bile Microscopy*

Bile duct biles were also examined for biliary crystals by light microscopy under direct and polarised light. Cholesterol crystals were scant, being observed in very small numbers in three bile samples only. Alternatively, calcium bilirubinate was the most common precipitate present and was observed in nine bile duct bile samples.

### *PBDS Bile Culture and Microscopy*

A relationship was observed between bile duct stones that were defined as primary, stones that formed de novo in the bile ducts, and moderate to profuse infection of the associated bile duct bile and excess precipitation of calcium bilirubinate in it. A combination of PBDS, positive bile culture and excess precipitate of calcium bilirubinate in bile duct bile was observed in five patients, two of whom had not had a previous cholecystectomy.

### DISCUSSION

This report presents investigative results on the morphological and chemical features of primary bile duct calculi. These are those calculi that have been reported to occur usually two years post cholecystectomy[5]. Early observations by Harding Rains (1964)[19] have suggested that these calculi may result via the secondary deposition of soft cholesterol and pigment layers on bile duct concretions, thereby giving rise to "soft" stones with a friable character.

Examination of the gross morphology of "soft" calculi obtained from the bile ducts of 27 patients, suggested that such calculi had unique morphological features not observed in gallstones. Most evident was a fragile structure with shapes that conformed to the contours of the bile duct. Further, they had an "earthy" texture and a high level of biliary pigmentation. All of these bile duct stones were observed to be brown or dark brown in colour. They consisted of alternating light and dark brown laminated layers on cross-section. The central area was always pigmented, giving the impression that stone formation had occurred by the gradual deposition of pigmented layers upon an initial nucleus of diffuse pigment.

It has been claimed that the identification of PBDS by gross morphology at the time of surgery can be difficult. Furthermore, if the gallbladder is still *in situ* or has only recently been removed, origin of the stone in the bile duct is difficult to prove. Based on these concepts, a number of logical criteria have been established that could define a PBDS, namely a previous cholecystectomy, a two year asymptomatic period after cholecystectomy, bile duct calculi with morphological appearances of PBDS and no evidence of a bile duct stricture or a long cystic duct remnant[5].

That such criteria can define PBDS there can be no doubt, since this is supported by a number of reports which have made similar claims[4,20,22]. However, Madden (1978)[9] has suggested that such patient related criteria may limit the occasions in which PBDS can be identified. Hence, following extensive studies into PBDS structure, Madden (1968, 1973)[7,8] has suggested that PBDS could be accurately identified by morphologic features alone. These included an "earthy" texture, easy fragmentation and with shapes that conformed to the contours of the bile duct. Madden (1978)[9] subsequently demonstrated the presence of PBDS in patients with *in situ* acalculus or calculus gallbladders.

This study has investigated bile duct stones from patients with and without a previous cholecystectomy. Approximately one-third of the patients thought to have PBDS were collected at a second operation in patients who had undergone a cholecystectomy 2–12 years previously. These patients with PBDS met the minimum requirements set out by Saharia and colleagues (1977)[5] for a positive diagnosis. The other two-thirds of patients with possible PBDS, had their bile duct stones collected at cholecystectomy. Twelve of these patients had an acalculus "normal" looking gallbladder, four had gallstones concomitantly present in the gallbladder and one patient had a gallbladder carcinoma. Collectively, the morphological and chemical properties of the bile duct stones from both surgical groups were similar, if not almost identical.

The results of this study support Madden's earlier work[9]. The PBDS in this series appear to occur irrespective of a previous cholecystectomy and whether or not the gallbladder contained any stones. Nevertheless one may still argue though that if there is an acalculous or calculous gallbladder present concomitantly with bile duct stones, the origin of the bile duct stones would logically follow to be in the gallbladder. Since gallstone disease in the gallbladder is a much more common occurrence than in the bile ducts. Nevertheless, the morphological and chemical profiles of what were presumed to be PBDS in the pre-cholecystectomy group of patients were consistent with current knowledge of "earthy" PBDS structure and composition[27,28] as well as being very similar to those PBDS from the post-cholecystectomy group of patients, namely, deeply pigmented "earthy" stones with shapes that adhere to that of the bile duct. As expected, PBDS composition was markedly different to the SBDS (Table 2). These latter calculi were gallstones that had migrated into the bile duct. They were of the multiple cholesterol variety, whereas in two other patients, brown pigment gallstones which have a composition similar to that of PBDS were observed, but with morphologies in terms of general shape and appearance that were different to that of PBDS.

Studies on PBDS composition have shown that these bile duct stones have low levels of cholesterol, less than 25% by weight in a study by Watts *et al* (1981)[11]. Bile duct stones that form de novo in the bile ducts also have high levels of bilirubin and calcium[10–12,27].

In comparing the bile duct stones from this series of patients with an array of gallbladder stones ranging from cholesterol single and multiple samples (approximately 80% of gallstone population at cholecystectomy)[18] to brown and black pigment gallstones, it was clear that PBDS in terms of gross morphology were distinct from all gallstones. On closer examination of their structure and cross section, PBDS were observed to belong to the brown pigment gallstone variety. Their cross sectional structural features of alternating light and dark brown pigment layers as well as their chemical compositions were observed to be similar[18]. This composition pattern was also partly observed in a single bile duct stone of one patient with a previous cholecystectomy. The core was observed to be "hard" and pale with a very high content of cholesterol (99%). The outer layer was alternatively "soft", fragile and highly pigmented. It consisted of 20% by weight of bilirubin and calcium. Infrared spectroscopy analysis showed that the calcium was present in the form of calcium bilirubinate and calcium palmitate, the salts of calcium that have been shown to predominate in PBDS. Hence, given that the outer shell fell away from the inner core and that their respective morphological and chemical features were so different, it was more than apparent that the outer layer had not been part of the original calculus. A gallstone appears to have migrated into the bile duct from the gallbladder, where "biliary mud" was then deposited, resulting in a gallbladder/bile duct combination stone.

Although Bernhoft *et al.* (1984)[27] claim that it is not possible to reliably identify primary bile duct stones on the basis of their appearance, our results disagree with this view. The results of this study have indicated that a range of brown pigment bile duct stones which were without doubt primary, and thus had formed de novo in the bile duct, had a characteristic morphology that correlated closely to a certain chemical composition profile. Extrapolating this relationship in a study of bile duct stones produced a positive correlation between a characteristic morphology and chemical composition for PBDS that went beyond the limits imposed by those criteria that have previously defined the de novo formation of stones in the bile duct. Thus a more detailed examination of PBDS morphology that can be supplemented by chemical analysis may well provide a basis from which a definite and more accurate diagnosis of PBDS might be made.

Bile duct bile associated with PBDS was observed to be infected, and on microscopic examination abundant calcium bilirubinate precipitation was present. Given that PBDS have a high concentration of calcium and bilirubin, bacterial activity in the form of the beta-glucuronidase mechanism may play a significant role in the initiation and growth of PBDS, which might be the prime factor in their aetiology.

### *References*

1. Aschoff, L. (1924) Lectures on pathology. Paul B. Hoeber: New York
2. Imamoglu, K., Perry, J.F. Jr., Root, H.D., Crisp, N.W., Jenson, C.H. and Wangensteen, O.H.

(1959) Formation of calculi following cholecystectomy attending partial occlusions of the common bile duct. *Surg. Forum* **9**, 521–525
3. Imamoglu, K., Yonehiro, E.G., Perry, J.F. Jr. and Wangensteen, O.H. (1958) Further experimental observations on the role of stasis of the extrahepatic biliary tract in the genesis of gallstones. *Surg. Forum* **8**, 225–229
4. Bartlett, M.K. (1972) Retained and recurrent common bile duct stones. *Amer. Surg.* **38**, 63–68
5. Saharia, P.C., Zuidema, G.D. and Cameron, J.L. (1977) Primary common bile duct stones. *Ann. Surg.* **185**(5), 598–604
6. Orr, K.B. (1980) Suture material as a nidus for formation of common bile duct stones. *Aust. NZ. J. Surg.* **50**(5), 493–494
7. Madden, J.L., Vanderheyden, L. and Kandalaft, S. (1968) The nature and significance of common duct stones. *Surg.Gynec.Obstet.* **126**(1), 3–8
8. Madden, J.L. (1973) Common bile duct stones. Their origin and management. *Surg. Clin. Nth. Amer.* **53**(5), 1095–1113
9. Madden, J.L. (1978) Primary common bile duct stones. *World J.Surg.* **2**, 456–471
10. Nayman, J., Vitetta, L., Sali, A. and Hocking, C. (1981) Primary bile duct stones. *Aust.NZ. J. Surg. Res. Proc.* **52**, 280–281
11. Watts, J.Mc.K., Whiting, M.J., Bradley, B. and Jarvinen, V. (1981) The chemical composition of common bile duct stones. *Proc.3rd Annual Meeting Int.Bil.Assoc.*, p16
12. Sali, A., Vitetta, L., Hocking, C., Kune, G.A. and Nayman, J. (1982) The composition of primary bile duct stones. *Proc.7th World Congress Coll. Chir. Dig.* (Tokyo) F018
13. Roschlau, P. (1974) Test combination cholesterol. Catalase method (Boehringer-Mannheim). *Z. Klin. Chem. Klin. Biochem.* **12**, 403
14. Henry, R.J., Cannon, D.C. and Winkelman, J.W. (1974) Clinical chemistry principles and techniques. Harper & Rowe, ch. 22 p. 1051
15. Nakayama, F. (1968) Quantitative microanalysis of gallstones. *J. Lab. Clin. Med.* **72**(4), 602–611
16. Soloway, R.D., Trotman, B.W. and Ostrow, J.D. (1977) Pigment Gallstones. *Gastroenterol* **72**, 167–182
17. Masuda, H. and Nakayma, F. (1979) Composition of bile pigment in gallstones and bile and their etiological significance. *J. Lab. Clin. Med.*, **93**, 353–360
18. Vitetta, L., Sali, A., Chou, S.T., Fleming, H., Little, P. and Elzarka, A. (1988) Gallstones at Autopsy and Cholecystectomy. *Aust. NZ. J. Surg.* **58**(7), 561–568
19. Sutor, D.J. and Wilkie, L.I. (1977) The crystalline salts of calcium bilirubinate. *Clin. Sci. Mol. Med.* **53**, 101–103
20. Henichart, J.P., Bernier, J.L., Roman, M. and Roussel, P. (1982) Identification of calcium palmitate in gallstones by IR spectroscopy. *Clin. Chim. Acta.* **118**, 279–287
21. Capocaccia, L., Ricci, F., Angelica, F., Angelica, A. and Attili, A.F. (1983) Proc. International Workshop on Epidemiology and Prevention of Gallstone Disease. MTP Press Ltd.
22. Orloff, M.J. (1978) Importance of surgical technique in prevention of retained and recurrent bile duct stones. *World J.Surg.* **2**, 403–410
23. Nayman, J. (1978) Do primary common bile duct stones occur in the duct? *Aust. NZ. J. Surg.* **48**(2), 189–190
24. Colcock, B.P. and Liddle, H.V. (1958) Common bile duct stones. *New Engl. J. Med.* **258**(6), 264–268
25. Cetta, F. and DeMauro, F. (1983) Chemical, mineralogical and microbiological differences between retained and recurrent common bile duct stones. Proc. 30th Congress International Soc. of Surgery, Hamburg. p. 104
26. Whiting, M.J., Watts, J.Mc.K. (1986) Chemical composition of common bile duct stones. *Br. J. Surg.* **73**, 229–232
27. Bernhoft, R.A., Pelligrini, C.A., Notson, R.W. and Way, L.W. (1984) Composition and morphologic and clinical features of common duct stones. *Am. J. Surg.* **148**, 77–85
28. Vitetta, L., Sali, A. and Nayman, J. (1986) Is the aetiology of primary choledocholithiasis unique? *Coll. Int. Chir. Dig. 9th World Congress* **126**

*(Accepted by M.Little 15 January 1991)*

### INVITED COMMENTARY

This interesting report by Vitetta and colleagues correlates the morphological characteristics with the physico-chemical analysis of stones removed from the bile ducts. Twenty seven of 42 bile duct stones studied here were soft, brown and conforming to the shape of the bile duct and presumed to be primary common bile duct stones. The others were assumed to be secondary to gall bladder stone formation. There is good supporting evidence for this assumption in this paper. However, this may not be the only method of primary bile duct stone formation and secondary stones may grow further once they have migrated into the bile duct and appear similar to primary stones. The morphological appearance of the soft brown pigment stone is related to the method of its formation with layer on layer of calcium bilirubinate and calcium palmitate. The bacteria, *E. coli, klebsiella* and bacteroides are frequently associated with soft brown bile duct stones[1]. These bacteria are known to produce beta-glucuronidase which converts the soluble bilirubin diglucuronide into the insoluble monoglucuronide and the bile duct moulds the stone into an ovoid shape[2].

Other methods of primary bile ducts stone formation have been proposed. In the prairie dog soft brown stones can be induced by a high calcium diet without the presence of bacteria but whether a high oral calcium diet causes an increased bile calcium concentration in humans is uncertain[3]. Small dark bilirubinate stones develop as a consequence of poor liver function in the absence of infection. While most occur in the gall bladder, some are widely distributed in the intra hepatic ducts. Thus, if all primary bile duct stones are regarded as "a priori" stasis stones associated with sepsis, other factors may be overlooked in these less common situations.

The soft brown bile duct stone appears to develop as a consequence of bacterial overgrowth. It has shown to have distinct physical and chemical characteristics which allow its recognition in the operating theatre and in the laboratory. But it would be better to classify it as a bile duct "Stasis Stone" rather than a "primary bile duct stone". Then, having such a classification begs the question of "What is the cause of the stasis"? Is the stasis due to something within the duct (stone or parasite)? Is the stasis due to a sphincter abnormality (duodenal diverticulum, stenosis, motility disorder, abnormal pancreaticobiliary union)? Is the stasis due to an abnormality of the duct (stricture, Caroli's disease, polycystic disease of the liver, choledochal cyst)[4]? In some of these cases the cause of the stasis will be obvious and indicate corrective surgical treatment. In others, particularly sphincter of Oddi dysfunction, the diagnosis may only be made with specific investigations. This paper is a valuable aid to the clinician to enable classification of stasis stones both morphologically and chemically to justify either investigation or surgical treatment of the cause of stasis.

### *References*

1. Leung, J.W., Sung, J.Y. and Costerton, J.W. (1989) Bacteriological and electron microscopy examination of brown pigment stones. *J. Clin. Microbiol.* **27**, (5), 915–921
2. Skar, V., Skar, A.G. and Stromme, J.H. (1988) Beta-glucuronidase activity related to bacterial growth in common bile duct bile in gallstone patients. *Scand. J. Gastroenterol.* **23**, (1), 83–90

3. Magnuson, T.H., Lillemoe, K.D., Peoples, G.E. and Pitt, H.A. (1989) Oral calcium promotes pigment gallstone formation. *J. Surg. Res.* **46**, (4), 286–291
4. Nakanuma, Y., Terada, T., Nagakawa, T., Kakita, A., Yoshikawa, T., Ohta, G., Yamaguchi, K. and Yakamoto, K. (1988) Pathology of hepatolithiasis associated with biliary malformation in Japan. *Liver* **8**, (5), 287–292

Ross Smith
Department of Surgery,
Royal North Shore Hospital,
Pacific Highway,
St. Leonards 2145,Sydney,
Australia

